# Design, Synthesis and Biological Evaluation of Some Triazole Schiff’s Base Derivatives as Potential Antitubercular Agents

**DOI:** 10.2174/1874104501812010048

**Published:** 2018-04-30

**Authors:** Asma A. Sager, Zainab S. Abood, Wedad M. El-Amary, Salah M. Bensaber, Inass A. Al-Sadawe, Nouri B. Ermeli, Salah B. Mohamed, Mohamed Al-Forgany, Ibrahim A. Mrema, Mabrouk Erhuma, Anton Hermann, Abdul M. Gbaj

**Affiliations:** 1 National Medical Research Centre, Zawia, Z16, Libya; 2 Department of Natural Products, Faculty of Pharmacy, University of Tripoli, Tripoli, Libya; 3 Department of Medicinal Chemistry, Faculty of Pharmacy, University of Tripoli, Tripoli, Libya; 4 National Center of Infectious diseases, Tripoli, Libya; 5 Department of Cell Biology & Physiology, Division of Cellular and Molecular Neurobiology, University of Salzburg, Salzburg, A-5020, Austria

**Keywords:** Schiff base, Triazole, Imines, Antitubercular activity, Thiolactomycin analogues, KasA enzyme inhibitors

## Abstract

**Background:**

Tuberculosis (TB) is the second important cause of death worldwide caused by a bacterium called *Mycobacterium tuberculosis*. There is a need to find and develop new Anti-TB medications that are effective, inexpensive and suitable with human immunodeficiency virus and other anti-TB drugs used in many countries and mainly the developing countries where the disease is widespread. These drugs must be designed to shorten treatment time and to be active against resistant forms of the mycobacteria that will help to increase the patients compliance. A key compound which could be used as a lead to meet these requirements, is the thiolactomycin (TLM). This antibiotic which is naturally available has an ability to treat *M. tuberculosis* by inhibiting condensing enzymes called FAS II (mtFabH, KasA and KasB) which are related to biosynthesis of mycolic acid.

**Methods:**

Our main aims are to design and synthesize analogues of TLM as new lead molecules which could be a possible anti–TB candidate. To overcome the synthetic challenges associated with preparing the chiral TLM analogues; we synthesized and investigated a series of triazole analogues as inhibitors of KasA enzyme and the whole cell *Mycobacteria*. A series of twelve compounds were synthesized, purified and fully characterized using several spectroscopic techniques. Molecular modelling studies for our synthesised compounds were achieved by using a modelling program called AutoDock 4.2 utilising rigid docking.

**Results:**

Our results indicate that analogues of TLM show a good activity as compared to TLM.

**Conclusion:**

The activity obtained for the synthesized compounds against *Mycobacteria tuberculosis* indicate that the synthesised compounds **1**, **2**, **6** and **9** are pharmacologically active as they restrained the growth of the *Mycobacteria* bacteria.

## 1 INTRODUCTION

Tuberculosis (TB), caused by the *Mycobacterium tuberculosis*, is an airborne infectious disease that is preventable and curable, but at the same time, if it is not well controlled, it can be fatal [1]. One third of the world's population which is about two billion people are thought to be infected with *M. tuberculosis* and one in every ten of those people will become sick with active TB in their lifetime [2-4]. People who acquired HIV are at an even greater risk. The developing of multidrug-resistant for *Mycobacterium tuberculosis* strains has hampered the efforts to control the disease. Effective control of the disease requires the development of novel drugs and the identification of new drug targets [5]. The cellular envelope of *M. tuberculosis* contains a peptidoglycan layer, free lipids, and a polypeptide layer. In addition to the cellular envelope, there are also mycolic acids which are a complex structure of fatty acids [6] which are the main constituents of the mycobacterial cell wall and may have a role as an efficient lipophilic obstacle to the diffusion of some known antibiotics through the cell wall. Taking into consideration, the significance of mycolic acids for mycobacteria growth and the enzymes that are involved in the metabolism of mycolic acids signify attractive targets for the designing of novel anti-mycobacterial agents [5].

**Fig. (1):**
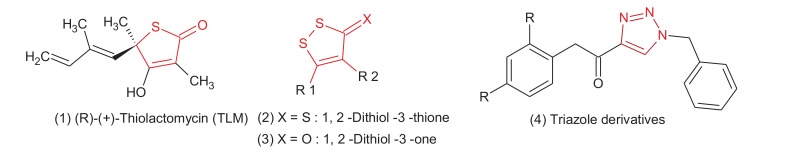
Chemical structure of thiolactomycin, 1, 2-dithiol-3-thione (2) and (3)1, 2-dithiol-3-one.

KasA, the mycobacterial β-ketoacyl ACP synthase I, is an important key enzyme within the FAS-II system. The enzyme catalyzes the condensation between malonyl-AcpM and the growing acyl chain *via* a ping-pong mechanism [7, 8]. KasA has been shown to be essential in *Mycobacteria* since conditional depletion of KasA induces cell lysis. These results emphasize the importance of KasA in *Mycobacteria* and suggest that this enzyme may be considered as an attractive target for novel drug development against *M. tuberculosis* [8-13].

(R)-(+)-Thiolactomycin (TLM, Fig. **1**), a distinctive thiolactone antibiotic isolated initially from a soil *Nocardia* specious,which is able to inhibit the mycobacterial β-ketoacyl synthases KasA and KasB, but for KasA is being the most sensitive. TLM shows potent **in vivo** activity against many pathogenic bacteria, including Gram-positive, Gram-negative bacteria and *M. tuberculosis* [7, 8]. Recent kinetic studies revealed that TLM can bind to both the acylated form of KasA and the free enzyme. Furthermore, TLM preferentially binds to the intermediate of the acyl-enzyme with a slow rate of binding kinetics throughout the inhibition reaction, which has a vital role for the **in vivo** activity of the compound [12].

Many studies have synthesised and tested TLM analogues, these analogues have aliphatic and other substituents which are attached to the 5-position of a thiolactone intermediate and the activity was significantly enhanced against the synthesis of the mycolate. Good results were achieved with ten carbon isoprenoid-based side-chains [5]. Nevertheless, the use of substituents with expected conformation and known chemical stability could be useful in getting potential drugs and help in understanding their mechanism of action. Over the last 25 years, TLM has been largely investigated as an excellent lead molecule for drug development in TB through its activity against KasA and KasB enzymes in fatty acid biosynthesis. However, the main disadvantage of this molecule which limited its use as TB drug is the chirality of the C_5_ carbon atom of the thiolactone ring [14, 5, 8]. Generally, single enantiomers are preferred in modern drug discovery to limit the risk of unwanted side effects and undesirable pharmacokinetics and pharmacodynamics upon the use of the racemic mixture of any drug [5]. A search of the literature identified few achiral rings that could be considered as isosteric substitutes for the chiral thiolactone ring of TLM, and which have shown interesting activities as FabH inhibitors. The synthesis and biological investigation of 1,2-dithiol-3-thione (2) and 1,2-dithiol-3-one (3) derivatives (Fig. **1**) as achiral analogues of TLM were reported to exhibit antimycobacterial activity [15]. Regarding with the cytotoxic activity, similar 1,2,4-triazole derivatives have been synthesized and biologically evaluated recently by many research groups as antimycobacterium tuberculosis agents and they found that the cytotoxicity and **in vivo** pharmacokinetic parameters have an acceptable safety index, **in vivo** stability and bio-availability [16-18].

## 2 METHODS

### 2.1 Materials and Solvents

All chemical solvents and materials utilised in the chemical synthesis of Schiff bases were of highest purity and were utilised with no further purification and they were purchased from Fluka analytical company (UK), and Sigma-Aldrich chemical company (UK). Standard isoniazid was purchased from Sigma-Aldrich (Sigma Chemical Company, St.Louis, MO, USA) and was dissolved in sterile millipore water to the required drug concentrations.

### 2.2 Instruments

Microwave closed system (Milestone start E 2450 MHz, Bergamo, Italy) was used in the synthesis of the compounds pre-coated aluminium plates (silica gel 60778, Fluka) were used to perform the Thin Layer Chromatography (TLC). The resulted TLC spots were visualized with both long (366 nm) and short (254 nm) UV light. Melting points were determined using open capillary tubes on an Electrothermal SMP30 melting point apparatus (Stuart, UK). The infra-red spectra of the synthesized compounds were recorded in region 4000 - 400 cm^-1^ by Varian FT-IR spectrophotometer 660. In addition, the UV-visible spectra were recorded in 1cm quartz cuvette by Cary 5000 UV\VIS\NIR spectrophotometer. Proton and carbon NMR spectra were determined by Bruker Avance 400 MHz NMR Spectrometer (France) in DMSO using tetramethylsilane as an internal standard.

### 2.3 Isolation of Bacteria

*M. tuberculosis* was obtained from patients at the National Center of Infectious diseases, Tripoli, Libya. Bacterial cultures were kept at -80 °C in Middlebrook 7H9 broth with 0.025% Tween 80 (Becton Dickinson, Sparks, MD, USA) and 10% oleic acid albumin dextrose catalase, henceforth this full recipe will be called “medium,” and certain volumes were thawed for every study. To investigate the exponential-phase growth; all cultures were incubated in five percent carbon dioxide at 37°C in the medium for four days.

### 2.4 General Procedure for the Synthesis of Schiff's Base Derivatives Synthesis [19, 20]

The procedure used for the synthesis of Schiff`s base derivatives using microwave-synthesis, is described in the literature [19, 20]. Weight and mix equimolar amounts of the starting materials (3-amino-1,2,4 triazole and aldehyde) in small vials and put them in the microwave. Time and power of microwave were adjusted starting with 1 minute and 300 Watt. The progress of the reaction was monitored at time intervals using TLC. The optimum time and power to achieve each compound synthesized using this procedure are specified below. Pure products were achieved after suitable workup.

#### 2.4.1 3-[(*E*)-(4*H*-1,2,4-Triazol-3-ylimino) Methyl] Naphthalen-2-ol (1)

Compound **1** has been synthesized according to the general procedure by reacting 4H-1,2,4-triazol-3-amine (0.5 g, 11.89 mmol) with 2-hydroxynaphthalene-1-carbaldehyde (3) (1.62 g, 11.89 mmol) at 350 Watt for 2 min. The reaction was monitored by TLC using hexane and ethyl acetate (6:4) as a mobile phase, and detected with UV light at 254 nm. Pure crystals of compound **1** were achieved by three recrystallization steps of the crude product from ethanol. The purity of the achieved crystals was confirmed by TLC using the same mobile phase. (**Y** = 98%. **M. P** = 270.7 – 270.9 ^o^C. **FT-IR** (cm-1): 1528 cm ^-1^ (C=N), 2978 (C-H aromatic), 3284 (N-H str). **^1^H-NMR** (400 MHz, DMSO-d6): δ [ppm] 8.53 (s, 1H, HC=N), 8.27 - 7.35 (m, 6H, Ar-H), 6.24 (s, 1H, N-CH=N). **^13^C-NMR** (400 MHz, DMSO-d6): δ [ppm] 159 (HC=N), 156 (C=C*-OH), 153 (N-CH=N), 150 ((N)_2_ -C=N), 140.1(1C, Ar), 128.15(1C, Ar), 126.08 (2C, Ar), 122.10 (2C, Ar), 115.93 (1C, Ar), 111.34 (2C, Ar).

#### 2.4.2 *N*,*N*-Dimethyl-4-[(*E*)-(4*H*-1,2,4-triazol-3-ylimino)methyl] Aniline (2)

Compound **2** has been synthesized according to the general procedure by reacting 4H-1,2,4-triazol-3-amine (0.5 g, 11.89 mmol) with 4-dimethylaminobezaldehyde (0.887 g, 11.89 mmol) at 400 Watt for 2 min. The reaction was monitored by TLC using hexane and ethyl acetate (7:3) as a mobile phase and then detected with UV- light at a wavelength (254 nm). The pure crystals of compound **2** were achieved by three recrystallization steps of the crude product from ethanol.

The purity of the achieved crystals was confirmed by TLC using the same mobile phase. (**Y** = 96%. **M. P** = 203.2 – 203.4 ^o^C. **FT-IR** (cm-1): 1540 cm ^-1^ (C=N), 2893 (C-H aromatic), 3091 (N-H str). **^1^H-NMR** (400 MHz, DMSO-d6): δ [ppm] 9.55 (s, 1H, HC=N), 7.51 - 6.85 (dd, 4H, Ar-H), 6.24 (s, 1H, N-CH=N), 3.14 (s, 6H, (CH3)_2_-N). **^13^C-NMR** (400 MHz, DMSO-d6): δ [ppm] 159 (HC=N), 153 (N-CH=N), 150 ((N)_2_ -C=N), 127.06 (2C, Ar), 113.58 (2C, Ar), 43.33 (2C,(CH3)_2_-N).

#### 2.4.3 1-(4-Nitrophenyl)-*N*-(4*H*-1,2,4-triazol-3-yl) Methanimine (3)

Compound **3** has been synthesized according to the general procedure by reacting 4H-1,2,4-triazol-3-amine (0.5 g, 11.89 mmol) with 4-nitrobenzaldehyde (0.89 g, 11.89 mmol) at 450 Watt for 3 min. The reaction was monitored by TLC using hexane and ethyl acetate (8:2) as a mobile phase, and then detected with UV- light at wavelength (254 nm). Pure crystals of **3** were obtained by three recrystallization steps of the crude product from ethanol. The purity of the achieved crystals was confirmed by TLC using the same mobile phase. (**Y** = 98%. **M. P** = 268.7 - 269 ^o^C. **FT-IR** (cm-1): 1516 cm ^-1^ (C=N, str), 2914 (C-H aromatic, str), 3103 (N-H, str). **^1^H-NMR** (400 MHz, DMSO-d6): δ [ppm] 9.85 (s, 1H, HC=N), 8.92 - 8.33 (dd, 4H, Ar-H), 6.24 (s, 1H, N-CH=N). **^13^C-NMR** (400 MHz, DMSO-d6): δ [ppm] 164.37 (HC=N), 153.85 (N-CH=N), 150.22 ((N)_2_ -C=N), 151.73 (1C, Ar), 148.06 (1C, Ar), 127.66 (2C, Ar), 124.57 (2C, Ar).

#### 2.4.4 2-(((4H-1,2,4-Triazol-3-yl)Imino) Methyl) Phenol (4)

Compound **4** has been synthesized according to the general procedure by reacting 4H-1,2,4-triazol-3-amine (0.5 g, 11.89 mmol) with 2-hydroxybenzaldehyde (1.45 g, 11.89 mmol) at 450 Watt for 2 min. The reaction was monitored by TLC using hexane and ethyl acetate (5: 5) as a mobile phase, and then detected with UV- light at a wavelength (254 nm). Pure crystals of **4** were achieved by three recrystallization steps of the crude product from ethanol. The purity of the achieved crystals was confirmed by TLC using the same mobile phase. (**Y** = 96%. **M. P** = 263 .7 – 264.5 ^o^C. **FT-IR** (cm-1): 1512 cm ^-1^ (C=N), 2920 (C-H aromatic), 3194 (N-H str). **^1^H-NMR** (400 MHz, DMSO-d6): δ [ppm] 8.79 (s, 1H, HC=N), 7.83 - 7.12 (m, 4H, Ar-H), 6.24 (s, 1H, N-CH=N). **^13^C-NMR** (400 MHz, DMSO-d6): δ [ppm] 159.13 (HC=N), 164.34 (C=C*-OH), 153.23 (N-CH=N), 150.66 ((N)_2_ -C=N), 134.11(2C, Ar), 121.13(2C, Ar), 116.08 (1C, Ar).

#### 2.4.5 3-(((4H-1,2,4-Triazol-3-yl)Imino) Methyl) Benzene-1,2-diol (5)

Compound **5** has been synthesized according to the general procedure by reacting 4H-1,2,4-triazol-3-amine (0.5 g, 11.89 mmol) with 2,3-dihydroxybenzaldehyde (1.64 g, 11.89 mmol) at 400 Watt for 2 min. The reaction was monitored by TLC using hexane and ethyl acetate (7: 3) as a mobile phase and then detected with UV-light at the wavelength (254 nm). Pure crystals of **5** were obtained by three recrystallization steps of the crude product from ethylacetate. The purity of the achieved crystals was confirmed by TLC using the same mobile phase. (**Y** = 92%. **M. P** = 281 .7 – 282.5 ^o^C. **FT-IR** (cm-1): 1622 cm^-1^ (C=N), 2870 (C-H aromatic), 3275 (N-H str). **^1^H-NMR** (400 MHz, DMSO-d6): δ [ppm] 8.99 (s, 1H, HC=N), 7.33 - 7.02 (m, 3H, Ar-H), 6.24 (s, 1H, N-CH=N), 3.16 (s, 2H, OH). **^13^C-NMR** (400 MHz, DMSO-d6): δ [ppm] 159.98 (HC=N), 154.24 (C=C*-OH), 153.23 (N-CH=N), 150.66 ((N)_2_ -C=N), 148.57 (C=C*-OH), 128.61(1C, Ar), 122.46 (1C, Ar), 117.37 (2C, Ar).

#### 2.4.6 2-Ethoxy-4-[(*E*)-(4*H*-1,2,4-Triazol-3-Ylimino) Methyl]phenol (6)

Compound **6** has been synthesized according to the general procedure by reacting 4H-1,2,4-triazol-3-amine (0.5 g, 11.89 mmol) with 4-hydroxy-3-methoxybenzaldehyde (0.80 g, 11.89 mmol) at 350 Watt for 2 min. The reaction was monitored by TLC using hexane and ethyl acetate (8:2) as a mobile phase, and then detected with UV-light at wavelength (254 nm). The pure crystals of **6** were achieved by three recrystallization steps of the crude product from ethanol. The purity of the achieved crystals was confirmed by TLC using the same mobile phase. (**Y** = 95%. **M. P** = 151.2 – 151.6 ^o^C. **FT-IR** (cm-1): 1512 cm ^-1^ (C=N, str), 2633 (C-H aromatic, str), 2972 (N-H, str). **^1^H-NMR** (400 MHz, DMSO-d6): δ [ppm] 8.36 (s, 1H, HC=N), 7.52 - 6.94 (m, 3H, Ar-H), 6.24 (s, 1H, N-CH=N), 4.14 (q, 2H, CH2), 1.14 (t, 3H, CH3). **^13^C-NMR** (400 MHz, DMSO-d6): δ [ppm] 161.17 (HC=N), 153.85 (N-CH=N), 150.22 ((N)_2_ -C=N), 150.03 (1C, Ar), 148.00 (1C, Ar), 132.50 (1C, Ar), 125.06 (1C, Ar), 117.33 (1C, Ar), 110.76 (1C, Ar), 65.63 (1C, CH2), 17.35 (1C, CH3).

#### 2.4.7 1-Phenyl-*N*-(4*H*-1,2,4-triazol-3-yl) Methanimine (7):

Compound **7** has been synthesized according to the general procedure by reacting 4H-1, 2, 4-triazol-3-amine (0.5 g, 11.89 mmol) with benzaldehyde (0.63 g, 11.89 mmol) at 350 watt for 2 min. The reaction was monitored by TLC using hexane and ethyl acetate (7:3) as a mobile phase, and then detected with UV-light at the wavelength (254 nm). Pure crystals of **7** were obtained by three recrystallization steps of the crude product from ethanol. The purity of the achieved crystals was confirmed by TLC using the same mobile phase. (**Y** = 96%. **M. P** = 196.4 – 169.8 ^o^C. **FT-IR** (cm-1): 1590 cm ^-1^ (C=N, str), 3044 (C-H aromatic, str), 3241 (N-H, str). **^1^H-NMR** (400 MHz, DMSO-d6): δ [ppm] 8.36 (s, 1H, HC=N), 7.83 - 7.52 (m, 5H, Ar-H), 6.24 (s, 1H, N-CH=N). **^13^C-NMR** (400 MHz, DMSO-d6): δ [ppm] 158.17 (HC=N), 153.85 (N-CH=N), 150.22 ((N)_2_ -C=N), 137.17 (1C, Ar), 132.50 (1C, Ar), 130.24 (2C, Ar), 127.38 (2C, Ar).

#### 2.4.8 N-(Thiophen-2-Ylmethylene)-4H-1,2,4-Triazol-3-Amine (8)

Compound **8** has been synthesized according to the general procedure by reacting 4H-1,2,4-triazol-3-amine (0.5 g, 11.89 mmol) with thiophene-2-carbaldehyde (1.33 g, 11.89 mmol) at 350 Watt for 3 min. The reaction was monitored by TLC using hexane and ethyl acetate (8:2) as a mobile phase and then detected with UV- light at a wavelength (254 nm). Pure crystals of **8** were obtained by three recrystallization steps of the crude product from ethylacetate. The purity of the achieved crystals was confirmed by TLC using the same mobile phase. (**Y** = 91%. **M. P** = 166.7 – 167.8 ^o^C. **FT-IR** (cm-1): 1671 cm ^-1^ (C=N, str), 3128 (C-H aromatic, str), 3293 (N-H, str). **^1^H-NMR** (400 MHz, DMSO-d6): δ [ppm] 7.86 (s, 1H, HC=N), 7.57 - 7.22 (m, 3H, Ar-H), 6.24 (s, 1H, N-CH=N). **^13^C-NMR** (400 MHz, DMSO-d6): δ [ppm] 156.35 (HC=N), 153.85 (N-CH=N), 150.22 ((N)_2_ -C=N), 139.14 (1C, Ar), 131.24 (1C, Ar), 128.29 (1C, Ar), 126.93 (1C, Ar).

#### 2.4.9 1-(4-Chlorophenyl)-*N*-(4*H*-1,2,4-Triazol-3-yl) Methanimine (9)

Compound **9** has been synthesized according to the general procedure by reacting 4H-1,2,4-triazol-3-amine (0.5 g, 11.89 mmol) with 4-chlorobenzaldehyde (0.83 g, 11.89 mmol) at 350 Watt for 1 min. The reaction was monitored by TLC using hexane and ethyl acetate (6:4) as a mobile phase, and then detected with UV- light at a wavelength (254 nm). Pure crystals of **9** were obtained by three recrystallization steps of the crude product from ethanol. The purity of the achieved crystals was confirmed by TLC using the same mobile phase. (**Y** = 95%. **M. P** = 202.7 – 202.3 ^o^C. **FT-IR** (cm-1): 1570 cm ^-1^ (C=N, str), 2834 (C-H aromatic, str), 3140 (N-H, str). **^1^H-NMR** (400 MHz, DMSO-d6): δ [ppm] 8.55 (s, 1H, HC=N), 7.82 - 7.43 (dd, 4H, Ar-H), 6.24 (s, 1H, N-CH=N). **^13^C-NMR** (400 MHz, DMSO-d6): δ [ppm] 162.54 (HC=N), 153.85 (N-CH=N), 150.22 ((N)_2_ -C=N), 138.43 (1C, Ar), 133.06 (1C, Ar), 129.46 (2C, Ar), 126.77 (2C, Ar).

#### 2.4.10 N-((E)-3-Phenylallylidene)-4H-1,2,4-Triazol-3-Amine (10)

Compound **10** has been synthesized according to the general procedure by reacting 4H-1,2,4-triazol-3-amine (0.5 g, 11.89 mmol) with cinnamaldehyde (1.56 g, 11.89 mmol) at 600 Watt for 2.5 min. The reaction was monitored by TLC using hexane and ethyl acetate (5:5) as a mobile phase and then detected with UV- light at a wavelength (254 nm). Pure crystals of **10** were obtained by three recrystallization steps of the crude product from ethanol. The purity of the achieved crystals was confirmed by TLC using the same mobile phase. (**Y** = 93%. **M. P** = 157.2 – 158.0 ^o^C. **FT-IR** (cm-1): 1630 cm ^-1^ (C=N, str), 3264 (C-H aromatic, str), 3338 (N-H, str). **^1^H-NMR** (400 MHz, DMSO-d6): δ [ppm] 7.86 (s, 1H, HC=N), 7.61 - 7.37 (m, 5H, Ar-H), 7.10 – 6.96 (m, 2H, CH=CH, olefinic) 6.24 (s, 1H, N-CH=N). **^13^C-NMR** (400 MHz, DMSO-d6): δ [ppm] 164.13 (HC=N), 153.85 (N-CH=N), 150.22 ((N)_2_ -C=N), 138.11 (1C, Ar), 134.34 (1C, Ar-C*H=CH) 130.21 (2C, Ar), 129.24 (2C, Ar), 126.64 (1C, Ar), 114.72 (1C, Ar-CH=C*H).

#### 2.4.11 N-(Furan-2-ylmethylene)-4H-1,2,4-Triazol-3-Amine (11)

Compound **11** has been synthesized according to the general procedure by reacting 4H-1,2,4-triazol-3-amine (0.5 g, 11.89 mmol) with furan-2-carbaldehyde (0.98 g, 11.89 mmol) at 450 Watt for 1.5 min. The reaction was monitored by TLC using hexane and ethyl acetate (6:4) as a mobile phase, and then detected with UV-light at wavelength (254 nm). The pure crystals of **11** were obtained by three recrystallization steps of the crude product from ethanol. The purity of the achieved crystals was confirmed by TLC using the same mobile phase. (**Y** = 90%. **M. P** = 163.4 – 164.2 ^o^C. **FT-IR** (cm-1): 1610 cm ^-1^ (C=N, str), 2763 (C-H aromatic, str), 3010 (N-H, str). **^1^H-NMR** (400 MHz, DMSO-d6): δ [ppm] 8.12 (s, 1H, HC=N), 7.52 - 7.02 (m, 3H, Ar-H), 6.24 (s, 1H, N-CH=N). **^13^C-NMR** (400 MHz, DMSO-d6): δ [ppm] 153.85 (N-CH=N), 150.22 ((N)_2_ -C=N), 148.29 (HC=N), 146.27 (1C, Ar), 139.47 (1C, Ar), 115.45 (1C, Ar), 112.38 (1C, Ar).

#### 2.4.12 1-(4-Methoxyphenyl)-*N*-(4*H*-1, 2, 4-triazol-3-yl) Methanimine (12)

Compound **12** has been synthesized according to the general procedure by reacting 4H-1,2,4-triazol-3-amine (0.5 g, 11.89 mmol) with 4-methoxybenzaldehyde (0.80 g, 11.89 mmol) at 350 Watt for 2 min. The reaction was monitored by TLC using hexane and ethyl acetate (8: 2) as a mobile phase and then detected with UV-light at wavelength (254 nm). The pure crystals of **12** were obtained by three recrystallization steps of the crude product from ethanol. The purity of the achieved crystals was confirmed by TLC using the same mobile phase. (**Y** = 97%. **M. P** = 171.7 - 172 ^o^C. **FT-IR** (cm-1): 1520 cm ^-1^ (C=N, str), 2913 (C-H aromatic, str), 3144 (N-H, str). **^1^H-NMR** (400 MHz, DMSO-d6): δ [ppm] 9.23 (s, 1H, HC=N), 7.62 - 7.12 (dd, 4H, Ar-H), 6.24 (s, 1H, N-CH=N), 4.08 (s, 3H, CH3). **^13^C-NMR** (400 MHz, DMSO-d6): δ [ppm] 164.43 (1C, Ar), 163.28 (HC=N), 153.65 (N-CH=N), 150.01 ((N)_2_ -C=N), 131.67 (2C, Ar), 128.02 (1C, Ar), 115.46 (2C, Ar), 60.56 (1C, CH3).

### 2.5 Evaluation of Antimicrobial Activity

A colorimetric method was used to measure the susceptibility of *Mycobacterium tuberculosis* to antimicrobial agents. The procedure is based on an oxidation-reduction reaction of the dye and alamar blue as a growth indicator. This method is a rapid, quantitative and non-radiometric method and is largely preferred for the determination of antimicrobial susceptibility of *M. tuberculosis* [21-23]. DMSO control and isoniazid free control are also included in the study.

### 2.6 Procedure

All materials and equipments used were sterilized using autoclave and water ethanol mixture (30: 70). Inocula were prepared by growing strains of *M. tuberculosis* in 7H9 broth (Middlebrook 7H9 broth base [Difco, Detroit, Mich.]) supplemented with 22.5 µl tween 80 and 4.5 ml of 10% ADC supplement and adjusted to a turbidity equal to that of McFarland standard (No. 1), by using a blank (7H9 Middlebrook and BANTA) and diluting the culture 1:5 in broth. The synthesized compound has been prepared by taking the calculated weight of the compound adding a small amount of 2% DMSO and completing the volume with distilled water to give a final concentration equal to 5 mmol. Then, serial twofold dilutions of the synthesized compounds were prepared in 7H9 broth. An aliquot of 3.64 ml of drug containing media was taken and added to 0.36 ml of the bacterial suspension. The final concentration of mycobacteria in the susceptibility test tubes was ~ 6 × 10 ^5^ colony-forming unit (CFU)/ml. Control experiments were performed by using three tubes which were inoculated in the same manner. The entire tubes were incubated at thirty-five degree centigrade in ambient atmosphere. A test was performed for the control tubes after seven, ten and fourteen days to decide whether the drug-containing tubes were ready to be interpreted. This was accomplished by adding 0.05 ml of 5% tween 80 and 0.02 ml of alamar blue solutions to the control tube and incubated for two hrs at fifty degree centigrade. In case the colour of the control tube altered from blue to pink after two hours of incubation subsequently Tween and Alamar blue were added to the tubes that containing drug and then incubated for two hours at fifty degree centigrade.

### 2.7 Partition Coefficient

The lipophilicity of a drug is connected to its capability to cross cell membranes by means of passive diffusion. This property is typically expressed by the logarithm of the n-octanol/water partition coefficient, log Po/w. The log Po/w reveals the relative solubility of the drug in n-octanol (a model of the lipid-bilayer of a cell membrane) and water (the fluid inside and outside cells). Conventionally, log Po/w values are measured using the “shake-flask” with the n-octanol and water partition system. Partition coefficients (log p) were determined using ACD/logPdb^®^ v7.0 (Advanced Chemistry Development Inc., Toronto, Canada, 2003) software [24, 25].

### 2.8 Statistical Analysis

All experimental data were stated as mean ± SEM. The differences between the significance of treated samples and control samples were analyzed using one-way ANOVA. The level of significance was set at p-value less than 0.05. MIC (minimum inhibitory concentration, which is the lowest concentration of an antimicrobial that inhibits the visible growth of microorganisms after overnight incubation) was determined using linear regression method of the percent of mycobacteria cell *via*bility against the concentration of the tested compounds using Microsoft Excel software 2010.

### 3 RESULTS AND DISCUSSION

For the production of the test compound, isosteric substitution of the thiolactone ring was carried out in the TLM molecule with a smaller size and an easier to synthesize ring structure was carried out. Furthermore, the triazole ring which forms the main part of many drugs, has been studied and selected in this study. Depending on the simplicity and the flexibility of the synthetic route, we choose the synthesis of triazole derivatives containing the imine (Schiff`s base) functional group. Recent reports indicate the role of imine functionality in increasing the potency and reducing the toxicity of isoniazid and *p*-aminosalicylic acid [26, 27]. In addition, common structural features between TLM and the triazole imine derivatives are indicated in red colour in Fig. (**2**). These alterations were expected to behave in a similar manner at the active site of the target enzyme which we studied by molecular modelling.

### 3.1 Molecular Modelling Study

Two different free molecular modelling tools have been used to predict the interaction of the synthesized compounds, namely Discovery Studio Visualizer and AutoDock 4.2. The KasA enzyme was downloaded from the Protein Data Bank (PDB) (http://www.rcsb.org/pdb/results/results.do?tabtoshow=Current&qrid=58A7533). It has been reported that TLM binds to the malonyl binding pocket of KasA where the methyl groups carbon nine and carbon ten are positioned in two hydrophobic pockets formed by proline 280, glycine 318 and phenylalanine 402, phenylalanine 237, respectively. The intercalation of the isoprenoid tail into the space between two peptide bonds, mainly alanine279-proline280 and glycine403-phenyalanine404, additionally stabilizes this interaction. The O-1 oxygen and the nitrogen atoms of histidines, His311 and His345, in the active site have formed two strong hydrogen bonds [7]. All targeted compounds were docked into the KasA enzyme and the docking energy was determined. The docking results (Table **1**) show that most of the targeted triazoles fit and interact with the active site in a close manner to TLM with extra binding to the active site *via* the triazole moiety.

### 3.2 Chemistry

**Scheme 1:**
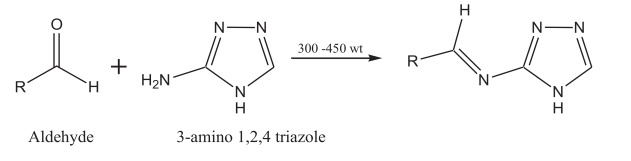
Synthesis of Schiff`s base from 3-amino-1,2,4-triazole and different aldehydes.

To generate a series of analogues with appropriate diversities that would allow us to obtain preliminary data on structure-activity relationships, our strategy was based on reacting 3-amino-1,2,4-triazole with different aldehydes to form imino (Schiff`s base) derivatives as described below (Scheme. **1**).

The R group could be a smaller or larger substituent and it can be of different nature either as electron withdrawing or electron donating groups (Table **1**). The purity of the achieved compounds was primarily checked by TLC using a suitable mobile phase. The infrared spectroscopic technique was used to confirm their chemical structures and purity. The mechanism of imine formation can be briefly explained as follows: the primary amine group of 3-amino-1,2,4-triazole combines with the desired aldehydes, protonation of the oxygen atom of the hydroxyl group is followed by a condensation reaction with elimination of water and the loss of the proton from the NH group. The amine group as strong nucleophilic group does not require acid catalysis for its initial addition to the carbonyl group. The IR spectrum of all synthsized compounds showed the disappearance of the carbonyl group which belongs to the aldehyde. At the same time, the amino group of the triazole which appears normally at 2500 – 3500 cm ^-1^, disappears in the IR spectrum of the purified product. The formation of the imine functionality was seen in the spectrum of the product by the characteristic peak which normally appears at lower wavelength. The ^1^H NMR spectra of the synthesized compounds were recorded in DMSO-d_6_, and the chemical shifts (δ), expressed in part per million (ppm) downfield from tetramethylsilane. The signals for the methene protons of the azomethine group, -N=C (H) were observed between 9.6 and 10.82 ppm, while the signals which represent the aldehyde proton completely disappeared in the same spectra.

### 3.3 Biology

As shown in the table below, the use of the oxidation-reduction dye Alamar blue gives an indication of the bacterial growth. Alamar blue is oxidized by metabolically active cells and its color is changed to pink to give maximum absorption (_max_) at 570 nm as in **3** and **7**, while in the case of dead cells, the dye will remain blue to give maximum absorption (λ_max_) at 600 nm as in the case of **2**, **6** and **9**. This gives us an indication that those compounds have an activity to inhibit the growth of *Mycobacterium tuberculosis*. Isoniazid, which is the most potent first line antitubercular agent, is used in this study as free control to ensure the sensitivity of the isolated microorganisms. The docking energies of all synthsized compounds are listed in the same table as well.

### 3.4 Structure -Activity Relationship (SAR)

**Fig. (3):**
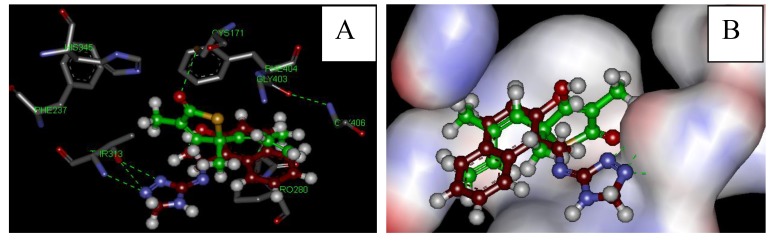
**(A)** Interaction of compound 1 (red) and TLM (green) at the active site of KasA. **(B)** Lipophilic interaction of compound 1 (red) and TLM (green) at the lipophilic pocket of KasA.

Depending on the biological and the docking results, we conclude that the activity of an effective drug is related to two main factors; the presence of a hydrophobic phenyl or naphthyl group at the R position, and the presence of atoms within the tested compounds that have the ability to form hydrogen bonds with the enzyme. The activity of the drugs will be abolished if the R group was aliphatic or when atoms to form hydrogen bonds were missing as in the case of **7**. The presence of an electron donating group at the *para* position will increase the activity 2, but the activity was abolished if the *para* position was occupied by an electron withdrawing group (**3**). If there is a halogen or a methoxy group in the *para* position this will increase the lipophilicity of the compound as it is the case with **9** and **12**, respectively. In **6** the activity is increased by the presence of a hydroxyl group at the *para* position which gives it the ability to form a hydrogen bond and the methoxy group which increases its lipophilicity. Comparison of the docking energy for synthesized compounds and TLM designated the possible increased interaction of the compounds under investigation as they need less binding energy as shown in Table (**1**). Compound 1 (red in Fig. **3a**) appears to be bound to the active site *via* three hydrogen bonds with THR 313, while the standard TLM (green in Fig. **3a**) forms a hydrogen bond with CYS171. In addition, the hydrophobic naphthyl group of compound 1 occupies the hydrophobic pocket, which lies between two peptide bonds (Ala279-Pro280 and Gly403-Phe404) mimicking the effect of the lipophilic isoprenoid moiety of TLM (Fig. **3b**). The latter may lead to further stabilization of the interaction at the active site and correspondingly increases the activity against the KasA enzyme.

### 3.5 Partition Coefficient Study

The partition coefficient has been widely used in calculating numerous physical properties such as membrane transport and water solubility. The partition coefficient of the tested compounds was determined to establish where a correlation between the lipophilicity and the MIC may be found. On one hand, the obtained results showed that compounds **1**, **2**, **6**, **9**, **12** had a log p values of +2.55, +2.22, +1.76, +2.5 and +1.81, respectively, which indicates strong lipophilicity and high anti-*Mycobacterium tuberculosis* activity. On the other hand, compounds **3**, **4**, **5**, **7**, **8**, **10** and **11** containing hydrophilic moieties on the aromatic ring showed lower log p values and showed no anti-*Mycobacterium tuberculosis* activity. The increase of lipophilicity at the aromatic rings as shown in Table (**1**) appears to greatly enhance cellular uptake of the compounds into the Mycobacteria and subsequently causes their extinction.

## CONCLUSION

The design and synthesis of some achiral TLM analogues were performed in order to replace the chiral thiolactone ring of TLM. The therapeutically important triazole ring scaffold was chosen to achieve the task. This choice was based on their structural similarities with the lead TLM and the availability of the chemicals and reagents required for use in our synthetic strategy. In addition, molecular modelling studies indicated that this type of TLM analogues may have good activity in comparison with the lead TLM. A series of twelve compounds were synthesized in good yield and purity using microwave technique. The achieved results indicate that compounds **1**, **2**, **6** and **9** are active as they inhibit the growth of *Mycobacteria*. The minimum of inhibitory concentrations of the active compounds is currently under investigation using the same procedure and serial dilutions of tested compounds. The preliminary structure – activity relationship based on the achieved results indicates the importance of the aromatic functionality for the activity. At the same time, electron donating groups and/or hydrogen bond donor moieties at the *para* position of the aryl group increase the activity. Once we have the acquired more data, interpretation of the emerging structure-activity relationships will help us to further develop new achiral TLM analogues as KasA inhibitors.

## Figures and Tables

**Fig. (2):**
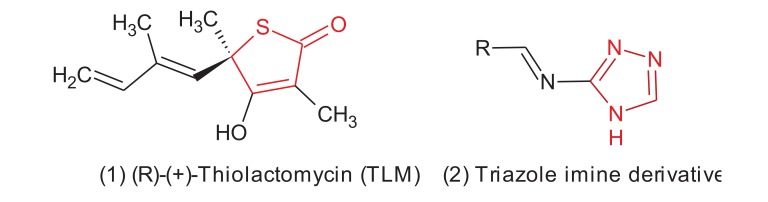
Chemical structure of TLM and triazole imine.

**Table 1:**
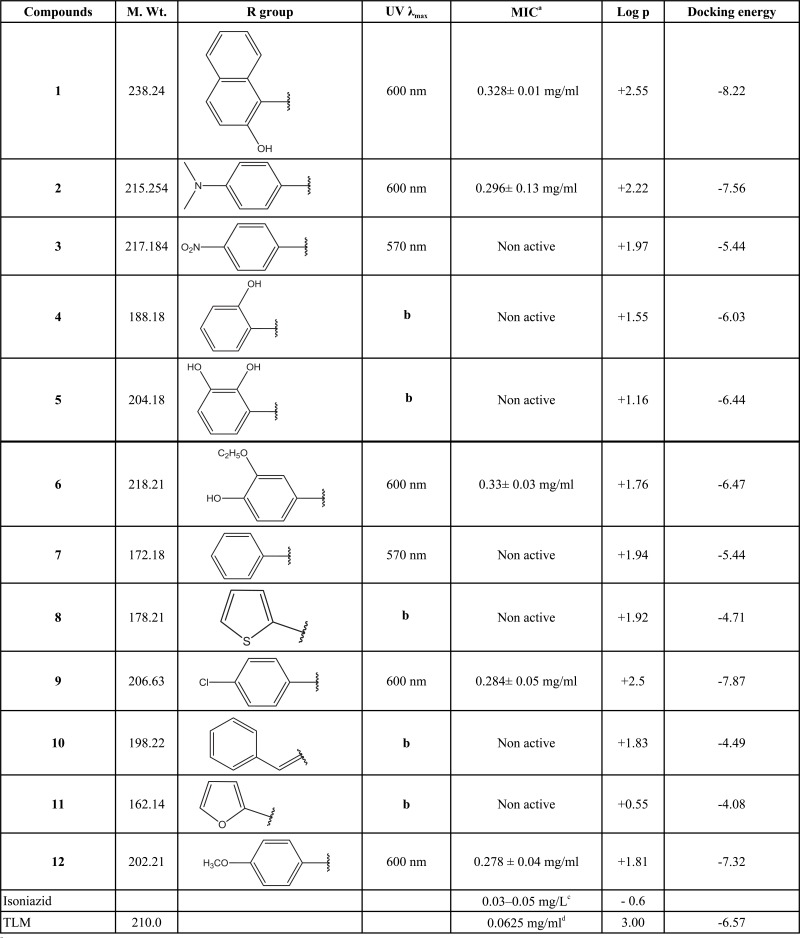
Biological evaluation of synthesized compounds against *Mycobacterium tuberculosis.*.

^a^Data are mean ± SD values. ^b^no change in the maximum absorption ^c^Literature reported MIC [28] ^d^ Literature reported MIC [29]
